# Remedying the Mitochondria to Cure Human Diseases by Natural Products

**DOI:** 10.1155/2020/5232614

**Published:** 2020-07-13

**Authors:** Jian-Kang Mu, Yan-Qin Li, Ting-Ting Shi, Li-Ping Yu, Ya-Qin Yang, Wen Gu, Jing-Ping Li, Jie Yu, Xing-Xin Yang

**Affiliations:** ^1^College of Pharmaceutical Science, Yunnan University of Chinese Medicine, 1076 Yuhua Road, Kunming 650500, China; ^2^Department of Pharmaceutical Preparation, The Xixi Hospital of Hangzhou Affiliated to Zhejiang University of Traditional Chinese medicine, Hangzhou 310023, China

## Abstract

Mitochondria are the ‘engine' of cells. Mitochondrial dysfunction is an important mechanism in many human diseases. Many natural products could remedy the mitochondria to alleviate mitochondria-involved diseases. In this review, we summarized the current knowledge of the relationship between the mitochondria and human diseases and the regulation of natural products to the mitochondria. We proposed that the development of mitochondrial regulators/nutrients from natural products to remedy mitochondrial dysfunction represents an attractive strategy for a mitochondria-involved disorder therapy. Moreover, investigating the mitochondrial regulation of natural products can potentiate the in-depth comprehension of the mechanism of action of natural products.

## 1. Introduction

As an important organelle in the cells, the mitochondria are considered the main powerhouse of the cells, because they can apply glucose, fatty acids, and certain amino acids as fuel sources to produce ATP through oxidative phosphorylation [1]. The mitochondria also play a critical role in many other processes, such as reactive oxygen species generation, maintenance of calcium homeostasis, adjustment of apoptotic cell death, regulation of lipid metabolism, and autophagy [2]. Thus, mitochondrial dysregulation of any form may lead to a variety of human diseases [2]. Mitochondrial dysfunction has been implicated in neurodegenerative disorders, cancer, liver diseases, myocardial injury, diabetes, and obesity [3, 4].

Natural products, including mixture and monomer, have been widely used to treat mitochondria-related diseases and have been reported as a highly significant source for the exploration of promising drugs/nutrients that have led to novel compounds for alleviating mitochondria-involved disorders, such as compounds with antitumor, neuroprotective, cardioprotective, hepaticprotective, antidiabetes, and antiobesity agents. The chemical synthesis of new drugs has rapidly developed in recent years with the advancement of combinatorial chemistry and computer-aided drug design technology [5]. However, due to the novel structures, therapeutic abilities, and certain unique pharmacological effects of the chemicals in natural products, the exploration of drugs and lead compounds from natural products is still an important approach for drug development [6].

The focus of this review was on mitochondrial regulation with natural products to treat human diseases. The purpose of this review was to examine the current knowledge of the relationship between mitochondria and human diseases and the regulation of natural products to the mitochondria. We proposed that the development of mitochondrial regulators/nutrients from natural products to remedy mitochondrial dysfunction represented attractive strategies for treating mitochondria-involved disorders. Moreover, investigating mitochondrial regulation of natural products can potentiate the in-depth comprehension of the underlying mechanism of action of natural products.

## 2. Remedying the Mitochondria to Cure Human Diseases by Natural Products

### 2.1. Regulating the Mitochondria to against Cancer

Prevention of cell death is a hallmark of human cancers and a major cause of treatment failure [7]. The mitochondria control the activation of apoptotic effects or mechanisms by regulating the translocation of proapoptotic proteins from the mitochondrial intermembrane space to the cytosol [8]. In addition, the mitochondria play an important role in various forms of nonapoptotic cell death and, especially, in necroptosis [7]. Because of their role in the regulation of basic cellular functions, it is not surprising that the mitochondria are involved in many aspects of tumorigenesis and tumor progression. For example, mutations in mitochondrial DNA that affect the compositions of the mitochondrial respiratory chain will lead to ROS overproduction, inefficient ATP production, and oxidative damage to the mitochondria and other macromolecules (including DNA), thus favoring chromosomal instability and carcinogenesis [9]. Furthermore, extensive polymorphisms and mutations in the mitochondrial DNA correlated with an increased risk of developing various malignancies [10]. Therefore, inducing cancer cells to undergo mitochondrial lesions and loss of function has become a very important direction in the field of anticancer drugs.

A large number of studies have shown that natural products have a significant anticancer activity by regulating the mitochondrial function with the following main mechanisms (Table 1): (1) promote the release of proapoptotic factors and induce tumor cell apoptosis by changes in mitochondrial membrane permeability, regulation of Bcl-2 family proteins, and other pathways; (2) regulate the mitochondrial energy metabolism, including the respiratory chain and tricarboxylic acid cycle; and (3) increase ROS levels and enhances oxidative damage.

### 2.2. Regulating the Mitochondria to against Neurodegenerative Diseases

Neurodegenerative diseases, such as Alzheimer's disease, Parkinson's disease, Huntington's disease, amyotrophic lateral sclerosis, and Friedreich's ataxia, are strongly age related and currently cannot be cured [11]. In neurons, efficient clearance of injured mitochondria through mitophagy plays a fundamental role in mitochondrial and metabolic homeostases and neuronal survival and health [11]. The mitochondria are organized in a highly dynamic tubular network that is continuously reshaped by opposing processes of fusion and fission [12]. Defects in fusion or fission will result in mitochondrial fragmentation, reduce energy metabolism, and increase oxidative stress, thus accelerating cell dysfunction and death, leading to neurodegenerative disease [13]. Therefore, the regulation of mitochondrial dynamics, such as fusion, fission, and mitochondrial phagocytosis, represents a significant avenue for controlling the fate of neurons [12, 13].

Through numerous animal experiments and clinical studies, a variety of drugs from natural products were identified with neuroprotective effects. Many of these drugs can exert neuroprotective effects by protecting the mitochondrial function (Table 2): (1) regulate *ΔΨ*m and membrane fluidity; (2) protect mitochondrial structure and morphology; (3) regulate mitochondrial apoptotic pathways, reduce the release of proapoptotic factors, and inhibit neuronal apoptosis; (4) improve the cellular mitochondrial respiratory function (energy metabolism); (5) enhance superoxide dismutase (SOD) activity, inhibit oxidative stress, and reduce ROS damage; and (6) improve mitophagy.

### 2.3. Regulating the Mitochondria to Remedy Liver Diseases

The liver, an organ with high energy requirements, plays a pivotal role in the synthesis and secretion of multiple endogenous compounds. Liver functioning is highly dependent on the mitochondria producing ATP for biosynthetic and detoxifying properties [14]. In previous studies, it was suggested that mitochondrial dysfunction is a critical factor in the initiation and progression of liver diseases, including ischemia/reperfusion (IR) injury, nonalcoholic/alcoholic fatty liver disease (NAFLD/AFLD), nonalcoholic/alcoholic steatohepatitis (NASH/ASH), and hepatic fibrosis, as well as intoxications by xenobiotics or heavy metals, bacterial, viral, and parasitic infections [15]. The mitochondria play an important role in the process of hepatic apoptosis and necrosis. The degree of the mitochondrial activity in the liver directly affects liver function [16].

In previous studies, it was shown that some natural medicines can protect liver cells from damage or liver fibrosis by protecting the mitochondrial function (Table 3): (1) stabilize the fluidity of mitochondrial membranes and protect the structure and morphology of liver mitochondria; (2) regulate the mitochondrial apoptotic pathway, reduce the release of proapoptotic factors, and inhibit hepatocyte apoptosis; (3) increase the mitochondrial energy metabolism; and (4) enhance SOD activity, inhibit oxidative stress, and reduce ROS damage.

### 2.4. Regulating the Mitochondria to against Diabetes and Its Complications

Diabetes mellitus (DM) is one of the most common metabolic diseases worldwide [17]. Patients with DM display hyperglycemia induced by a damage in insulin secretion (type 1), insulin action (type 2), or both. Type 1 diabetes mellitus (T1DM), which accounts for less than 10% of diabetes cases, is characterized by an immune-mediated destruction of *β* cells in the pancreatic islets of Langerhans, resulting in insulin deficiency [18]. Type 2 diabetes mellitus (T2DM), which accounts for less than 90% of diabetes cases, involves insulin resistance (IR) in peripheral tissues and increased levels of blood glucose, because of overnutrition with an insulin secretion defect [18, 19]. IR continuously exists in the development of T2DM. A defect in the secretion function of pancreatic beta-cell is the prerequisite of T2DM development [20]. Mitochondrial dysfunction is the common mechanism of IR and injury of secretion function of pancreatic beta-cell [20, 21]. Furthermore, many mitochondrial gene mutation sites related to diabetes have been found, and the 3243A → G mutation in the mtDNA tRNA^Leu(UUR)^ gene is the most common cause of mitochondrial diabetes [22]. This mutation results in the reduction of insulin release and insulin resistance and leads to persistent hyperglycemia, which in turn causes mitochondrial dysfunction and reduces insulin release [22]. Muscle biopsies of diabetic patients have revealed abnormal mitochondrial metabolism and reduced mitochondria quantity [23, 24].

A large proportion of the diabetic population develops chronic vascular complications leading to significant morbidity and mortality [25]. Microvascular complications include diabetic nephropathy, neuropathy, and retinopathy; muscle atrophy, coronary, and peripheral vascular diseases; and stroke [25]. The hyperglycemic milieu alters the epigenetic machinery and mtDNA. Other genes associated with mitochondrial homeostasis are epigenetically modified, thereby further contributing to mitochondrial damage [26]. Dysfunction is seen in the context of an altered mitochondrial metabolism and oxygen consumption, increased oxidative stress, and alterations to mitochondrial networking and turnover. An increasing body of evidence has highlighted the role of mitochondrial dysfunction in the development of diabetic complications [27, 28].

In previous studies, it was found that many natural products alleviated the symptoms of T2DM and its complications by protecting the mitochondrial function (Tables 4 and 5): (1) protecting the structure and morphology of the mitochondria from pathological organs/tissues; (2) regulating the mitochondrial apoptotic pathway, reducing the release of proapoptotic factors, and inhibiting cell apoptosis; (3) increasing mitochondrial energy metabolism; and (4) enhancing SOD activity, inhibiting oxidative stress, and reducing ROS damage.

### 2.5. Regulating the Mitochondria to Antiobesity

Obesity is caused by an imbalance between energy intake and expenditure and results in excessive energy that in adipose tissue is stored as triglycerides (TGs) [29]. It is not only recognized as a simple condition but also causes many metabolic diseases, such as cardiovascular disease, T2DM, hypertension, and fatty liver disease [30]. In many organs and tissues (including adipose tissue), the mitochondria are center stage in the control of energy homeostasis. Research evidence indicates that mitochondrial dysfunction in adipocytes is closely related to obesity [31]. Various physiological conditions, such as excessive nutrition and genetic factors, disrupt mitochondrial function by impairing mitochondrial biogenesis, dynamics, and oxidative capacity. Mitochondrial dysfunction in adipocytes may have impact on adipogenesis and insulin sensitivity and may significantly alter their metabolic function, which ultimately leads to obesity [32].

Animal experiments and clinical studies have successively identified many drugs from natural products for treating obesity. Many of these drugs can regulate mitochondrial function to treat obesity, primarily through promoting energy and fat metabolism (Table 6).

### 2.6. Regulating the Mitochondria to against Myocardial Injury

Myocardial injury can be caused by myocardial infarction, ischemia, inflammatory cell infiltration, poisoning, and so on [33]. The essence of myocardial injury refers to the edema, degeneration, and necrosis of myocardial cells; the breakdown and lysis of myofibrils; and cellular structures, such as mitochondria in severe lesions. Severe myocardial injury can lead to myocarditis and heart failure [34]. Myocardium is the most energy consuming tissue in the human body [35]. Mitochondrial abnormalities play a central role in the pathogenesis and development of various heart diseases, including acute myocardial infarction and cardiomyopathy [36].

In previous studies, it was shown that natural products can protect the heart by regulating the mitochondrial function (Table 7): (1) stabilize *ΔΨ*m and membrane fluidity; (2) protect mitochondrial structure and morphology; (3) adjust mitochondrial apoptotic pathways, reduce the release of proapoptotic factors, and inhibit myocardial cell apoptosis; (4) improve mitochondrial energy metabolism; and (5) enhance SOD and GSH activity, inhibit oxidative stress, and reduce ROS damage.

## 3. Similarities and Differences between the Mitochondrial Mechanisms for Natural Products Regulating Different Diseases

As shown in Table 8, there are some common mechanisms in mitochondrial dysfunction among different diseases, and the similarities and differences existed between the mitochondrial mechanisms for natural products regulating different diseases. For instance, almost all the mitochondria-involved diseases, including neurodegenerative disorders, cancer, liver diseases, myocardial injury, diabetes, and obesity, are related with mitochondrial energy metabolism, which can be remedied by natural products. However, fatty acid oxidation is specifically involved with obesity and fatty liver disease, which can also be regulated by natural products. Furthermore, a variety of natural products can remedy the mitochondria through multiple mechanisms to cure various diseases.

## 4. Conclusion

**Figure 1:**
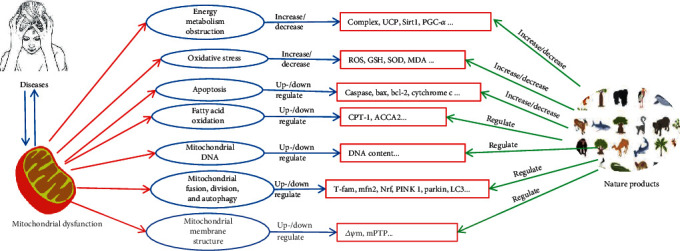
Remedying the mitochondria to cure human diseases by natural products.

Mitochondria are cytoplasmic organelles responsible for cell survival and cell death. Mitochondrial dysfunction has been reported to be involved in many diseases. Many natural products can regulate the mitochondria in various ways to alleviate related diseases (Figure 1). However, only a few have become clinical drugs for treating patients, and many compounds have not been used in clinical practice. Additional studies (such as pharmacodynamics, toxicology, and structure-activity relationship) of these compounds should be performed, which will promote that more natural products will be available for clinical usage. In addition, the monomers that can regulate the mitochondria in many natural extracts remain unclear, and further studies are warranted to identify natural monomers that can regulate the mitochondria. With the deepening of research, it is believed that more natural products that can regulate the mitochondria have the potential to be used in treating diseases, which is of utmost importance.

## Figures and Tables

**Table 1:** The anticancer activity of natural products.

Types of nature products	Natural products	Mitochondrial regulation	Types of cancers	Experimental models
Mixture	*Bulbine frutescens* [37]	Cell cycle arrest, ROS production, apoptosis induction, disruption of *ΔΨ*m	Triple negative and luminal breast cancer	Human breast cancer cells (MDA-MB-231 and T47D) and human embryonic kidney 293 (HEK293) cells
	Bullfrog oil [38]	Increases intracellular ROS levels, maintains DNA integrity, and reduces *ΔΨ*m	Melanoma	Human melanoma cells A2058

Monomer	Rhein [39]	Inhibits mitochondrial energy metabolism, decreases cellular ATP and ADP levels, changes the ratio of ATP to ADP, and induces mPTP opening	Liver cancer	Liver cancer cell lines (SMMC-7721 and SMMC-7721/DOX)
	Orientin [40]	Increases of intracellular ROS levels in HT29 cells in a dose-dependent manner, modulates Bcl-2 family proteins, induces mitochondrial cytochrome c release into the cytoplasm in a concentration-dependent manner	Human colorectal carcinoma	Colorectal carcinoma cells (HT29)
	Licochalcone A [41]	Increases the ratio of Bax/Bcl-2 and reduces the integrity of the mitochondria and promotes the release of cytochromes from mitochondria to the cytoplasm	Bladder cancer	Human bladder cancer cells (T24 and 5637)
	Asparanin A [42]	Induces apoptosis through the mitochondrial pathway, including the deregulation of Bak/Bcl-xl ratio, which leads to the generation of ROS, upregulation of cytochrome c followed by decrease of *ΔΨ*m, and activation of caspases	Endometrial cancer	Endometrial cancer cell line Ishikawa
	Parameritannin A-2 [43]	The combination of doxorubicin and parameritannin A-2 remarkably increases the release of cytochrome c and the activation of caspase-3 and caspase-9	Gastric cancer	HGC27 cells
	Gracillin [44]	Attenuates mitochondria-mediated cellular bioenergetics by suppressing ATP synthesis and producing ROS	Lung cancer	H1299, H460, and A549 cells
	Cernumidine [45]	The combination of cernumidine and cisplatin downregulates Bcl-2 and upregulates proapoptotic Bax and depletion of the *ΔΨ*m.	Bladder cancer	RT4, T24, and 5637 cells

**Table 2:** Neuroprotection activity of natural products.

Types of nature products	Natural products	Mitochondrial regulation	Types of diseases	Experimental models
Mixture	*Solanum melongena* extract [46]	Prevents apoptosis, reduces SOD, and increases ATP production and upregulates SOD and catalase activity	Rotenone-induced neurotoxicity	Rotenone-induced neurotoxicity in PC-12 cells
	*Ganoderma lucidum* [47]	Regulates *ΔΨ*m, radical oxygen species accumulation, and ATP depletion and activates the AMPK/mTOR and Pink1/Parkin signaling pathways	Parkinson's disease	MPTP- (1-methyl-4-phenyl-1,2,3,6- tetrahydropyridine-) induced mouse model

Monomer	Linalool [48]	Reduces mitochondrial ROS and calcium levels and maintains *ΔΨ*m to reduce oxidative stress	Glutamate-induced nerve injury	Glutamate-induced mitochondrial oxidative stress in immortalized neuronal HT-22 cells
	Cinnamic acid derivatives [49]	Blocks apoptosis and protects mitochondrial physiological functions	Neuroprotection and angiogenesis	H_2_O_2_-induced injury model in HBMEC-2 and SH-SY5Y cells
	Proanthocyanidins [50]	Inhibits signaling pathways involved in mitochondrial-mediated apoptosis	Methyl mercuric chloride-induced neurotoxicity	Cortical neuron cells from rats
	*α*-Arbutin [51]	Reduces oxidative stress, stabilizes *ΔΨ*m, and enhances adenosine triphosphate	Parkinson's disease	Rotenone-treated human neuroblastoma cells (SH-SY5Y) and drosophila Parkinson's disease model
	Naringenin [52]	Reduces oxidative load, which in turn maintains mitochondrial function and prevent neuronal cell death	H_2_O_2_-induced neurotoxicity	Human neuroblastoma SH-SY5Y cells
	Apigenin [53]	Reduces oxidative stress, downregulates the TLR4/NF-*κ*B signaling pathway, and inhibits mitochondrial-mediated neuronal apoptosis	Acrylonitrile-induced neuroinflammation	Acrylonitrile-induced neurotoxicity in rats
	Auraptene [54]	Enhances mitochondrial respiration and attenuates ROS production	Parkinson's disease-like behavior	Rotenone-treated SN4741 cells
	Naringenin [55]	Inhibits HO-induced mitochondrial dysfunction, including a decrease in membrane potential and Bcl-2/Bax ratio, cytochrome c release, and caspase-3 cleavage	H_2_O_2_-induced neurotoxicity	Human neuroblastoma SH-SY5Y cells
	Ulmoside A [56]	Induction of translocation of cytochrome-c, decrease of Bcl-2 level, increase of Bax level, and cleavage of caspase-3 in neuronal cells	Lipopolysaccharides- (LPS-) induced neurotoxicity	LPS-treated mouse neuroblastoma N2A cell line
	Celastrol [57]	Inhibits apoptosis of dopaminergic neurons by activating mitosis and degrading damaged mitochondria	Parkinson's disease	1-Methyl-4-phenylpyridinium- (MPP+-) induced SH-SY5Y cell model and MPTP-induced mouse model

**Table 3:** Liver protection activity of natural products.

Types of nature products	Natural products	Mitochondrial regulation	Types of liver diseases	Experimental models
Mixture	Rooibos tea [58]	Enhances the ability of the respiratory chain and energy production	Liver injury	Carbon tetrachloride- (CCl_4_-) induced liver damage in rats
	*Cimicifuga racemosa* extract [59]	Maintains mitochondrial integrity and ATP levels; prevents mitochondrial ROS formation, loss of *ΔΨ*m, and cell death; and mediates a switch from mitochondrial respiration to glycolysis	Liver injury	Erastin-treated HT22 cells and ras-selective lethal compound c-treated HepG2 cells
	Sipjeondaebo-tang [60]	Improves oxidative stress and regulate *ΔΨ*m	Liver injury	Iron/arachidonic acid-treated HepG2 and CCl_4_-induced acute liver injury in mice
	*Polygonatum kingianum* [61]	Inhibits the reduction of SOD, GSH, ATP synthase, and complex I and II, in the mitochondria; upregulates and downregulates mRNA expression of carnitine palmitoyl transferase-1 and uncoupling protein-2, respectively; inhibits the increase of caspase-9, caspase-3 and Bax expression in hepatocytes; and decreases the expression of Bcl-2 in hepatocytes and cytchrome c in the mitochondria	NAFLD	High-fat diet-induced NAFLD in rats
	*Punica granatum* L. [62]	Decreases the expression of uncoupling protein 2 (UCP2), restores the ATP content, inhibits mitochondrial protein oxidation, and improves mitochondrial complex activity in the liver	NAFLD	High fat diet-induced NAFLD in rats and ellagic acid treated HepG2 cells

Monomer	Betaine [63]	Enhances mitochondrial function by increasing mitochondrial fusion and improves cell survival	Liver injury	Oligomycin-/rotenone-treated human HCC (Huh7) cells
	Nicotinamide riboside [64]	Enhances Sirt1 and PGC-1*α* activity, reduces oxidative stress, and restores mitochondrial biogenesis and aerobic respiration	AFLD	Ethanol-induced AFLD in C57BL/6J mice and ethanol-treated HepG2 cells
	Puerarin [65]	Improves liver complex I and complex II activity and regulates mitochondrial DNA content	NAFLD	High-fat and sucrose diet-induced NAFLD in C57BL/6J mice
	Diosgenin [66]	Improves oxidative stress and increases *ΔΨ*m	NAFLD	Palmitic acid-induced NAFLD in L-02 cells
	Silybin [67]	Stimulates mitochondrial fatty acid oxidation, reduces basal and maximal respiration and ATP production in steatohepatitis cells, and rescues fatty acid-induced apoptotic signals and oxidative stress in steatohepatitis cells	NAFLD/NASH	Oleate/palmitate mixture and TNF*α*-treated rat hepatoma FaO cells
	Salvianolic acid B [68]	Decreases cytochrome c and caspase-3 protein expression, increases mfn2 mRNA expression and *ΔΨ*m, and enhances mitochondrial respiratory function	NASH	High-fat diet-induced NASH in rats
	NecroX-7 [69]	Reduces mitochondrial ROS and intracellular ROS/RNS levels, protects *ΔΨ*m, improves abnormal mitochondrial morphology, and reduces steatosis and oxidative damage by inhibiting mitochondrial ROS/reactive nitrogen species (RNS)	NASH	Leptin-deficient *ob/ob* and methionine/choline-deficient diet-fed *ob/ob* mice

**Table 4:** Anti-T2DM activity of natural products.

Types of nature products	Natural products	Mitochondrial regulation	Experimental models
Mixture	Polysaccharides from *Portulaca oleracea* L. [70]	Improves *ΔΨ*m, increases ATP production, depolarizes cell membrane potential, and increases intracellular Ca^2+^ levels	Tetrodotoxin-treated INS-1 cells
	Korean red ginseng [71]	Increases mtDNA copy number of mitochondrial biogenesis-related transcription factors (PGC-1*α* and T-fam)	*C57BL/KsJ db/db* mice (a genetic animal model of obese T2DM)

Monomer	Berberine [72]	Reduces mitochondrial ROS levels primarily through Sirt3 modification	Arsenic-induced Sirt3 modifications in isolated mitochondria from rat pancreas
	Quercetin [73]	Reduces ROS, increases complex I activity and electron transfer system coupling efficiency, increases cellular NAD/NADH ratio, and activates the PGC-1*α* mediated pathway	High-glucose-stimulated HepG2 cells
	Theaflavins [74]	Enhances the mitochondrial DNA copy number, downregulates the PGC-1 *β* mRNA level, and increases PRC mRNA expression	Palmitic acid-induced I/R in HepG2 cells
	Silibinin [75]	Improves mitochondrial quality, regulates *ΔΨ*m, and increases the Bcl-2/Bax ratio	Palmitic acid-induced apoptosis and mitochondrial dysfunction in pancreatic INS-1 cells
	Puerarin [76]	Improves the tricarboxylic acid cycle and oxidative phosphorylation function of the mitochondria of skeletal muscle, enhances the expression levels of regulators of mitochondrial biogenesis (Sirt 1 and PGC-1*α*), and increases the density of the mitochondria	High-fat diet-/streptozocin-induced diabetic rats and palmitate acid-treated rat L6 skeletal muscle cells

**Table 5:** Antidiabetic complications activity of natural products.

Types of nature product	Natural products	Mitochondrial regulation	Cured complications	Experimental models
Mixture	QiDiTangShen granules [77]	Improves mitochondrial quality and increases the expression of Sirt1 and the proportion of p-AMPK (thr172)/AMPK	Nephropathy	*db/db* mice
	Shengmai San [78]	Increases protein levels of complexes I, III, and V and regulates the activity of oxidative phosphorylation complexes I and IV	Cardiomyopathy	Leptin receptor-deficient *db/db* mouse and palmitate acid-treated H9C2 cells
	Water extracts of Liuwei Dihuang [79]	Improves *ΔΨ*m and inhibits NADPH oxidase activation, and ROS production	Muscle atrophy	Methylglyox-treated C2C12 myotubes and streptozocin-treated C57BL/6 mice

Monomer	Anthocyanins [80]	Inhibits the generation of ROS, cellular apoptosis, expression of cleaved caspase-3 and the Bax/Bcl-2 ratio and enhances the expression of cytochrome c released from mitochondria	Nephropathy	BKS *db/db* c57BL6 mice and high-glucose-stimulated HK-2 cells
	Orientin [81]	Regulates *ΔΨ*m and the activation of mitophagy	Nephropathy	High-glucose-treated MPC-5 cells
	Salidroside [82]	Increases mitochondrial DNA copy and electron transport chain proteins and improves the reduction of Sirt1 and PGC-1*α* expression	Nephropathy	Streptozotocin-induced diabetic nephropathy in obese mice
	Astragalus polysaccharides [83]	Inhibits the expression of proapoptotic proteins of both the extrinsic and intrinsic pathways and modulates the ratio of Bcl-2 to Bax in the mitochondria	Cardiomyopathy	High-glucose-stimulated H9C2 cells
	Ginsenoside Rb1 [84]	Reduces mitochondrial damage and activates oxygen production, enhances the Bcl-2/Bax ratio, and inhibits the expression of cleaved caspase-3 and cleaved caspase-9	Encephalopathy	Methylglyoxal-induced damage in SH-SY5Y cells
	Hydroxytyrosol [85]	Increases mitochondrial complex IV and HO-1 expression through activating the AMPK pathway, followed by preventing the high-glucose-induced production of ROS and reduces cell viability	Neuropathy	Male *db/db* C57BL/6J mice and SH-SY-5Y neuroblastoma cells

**Table 6:** Antiobesity activity of natural products.

Types of nature products	Natural products	Mitochondrial regulation	Experimental models
Mixture	Green tea [86]	Moderates CPT-1 and ACAA2 levels and reduces CPT-2 and ACAD levels	High-fat diet-induced obese in C57BL/6 mice
	Peanut sprout extracts [87]	Promotes mitochondrial fatty acid oxidation	Dibutyryl cyclic adenosine monophosphate- (cAMP-) stimulated 3T3-L1 cells and rosiglitazone-stimulated C3H10T1/2 cells
	Melinjo (*Gnetum gnemon* L.) seed extract [88]	Upregulates thermogenic uncoupling protein 1 (UCP1) and mitochondrial marker cytochrome c oxidase subunit IV protein expression in brown adipose tissue	High-fat diet-fed C57BL6J mice
	*Cinnamomum cassia* Presl [89]	Increases ATP levels by increasing the mRNA expression of mitochondrial biogenesis-related factors, such as PGC-1*α*, Nrf1, and T-fam	High-fat diet-induced obese mouse and mouse C2C12 myoblasts
	Guarana (*Paullinia cupana* Kunth) [90]	Increases the expression of PGC-1*α*, CREB1, AMPKA1, Nrf1, Nrf2, and Sirt1 in the muscle and brown adipose tissue and increases mtDNA (mitochondrial DNA) content in the muscle	High-fat diet-fed C57BL6J mice

Monomer	Isorhamnetin [91]	Regulates mitochondrial biosynthetic mRNA levels of PGC-1*α*, Nrf1, and T-fam and increases the mtDNA/nuclear DNA ratio	3T3-L1cells
	Zeaxanthin [92]	Increases mitochondrial DNA content and mRNA levels of genes related to mitochondrial biogenesis, reduces mitochondrial oxidative damage, improves *ΔΨ*m, and eliminates intracellular ROS and mitochondrial superoxide	3T3-L1 preadipocytes
	Berberine [93]	In a mouse model, protects mitochondrial structure and function by reducing ATP abundance and activity of complex I and enhances the activity of complexes II and IV. In a cellular model, decreases ATP abundance, increases *ΔΨ*m and inhibits apoptosis	High-fat diet-induced obese model in C57BL/6 mice with GLP-1 reduction
	Purpurin [94]	Regulates ROS and reduces *ΔΨ*m and ATP production	3T3-L1 murine preadipocytes and high-fat diet-fed C57BL/6 mice
	Epigallocatechin-3-gallate [95]	Increases the mtDNA content and the mRNA levels of PGC-1*α*, Nrf1, and T-fam in brown adipose tissue	High-fat diet-induced obesity in C57BL/6J mice

**Table 7:** Antimyocardial injury of natural products.

Types of natural products	Natural products	Mitochondrial regulation	Types of diseases	Experimental models
Mixture	Propolis [96]	Reduces the rate of H_2_O_2_ produced by mitochondrial respiration	Myocardial ischemia	Hypothermia-induced ischemia model in C57BL6J mice

Monomer	Capsaicin [97]	Inhibits the production of ROS, inhibits opening of the mPTP and activation of caspase-3, downregulates Bax, and upregulates Bcl-2	I/R injury	Acute myocardial hypoxia/reoxygenation (H/R) injury model in H9C2 cells
	Quercetin [98]	Increases cell viability, SOD, catalase, and GPx activity, GSH levels, *ΔΨ*m, and GSH/GSSG ratios and reduces LDH and caspase-3 activity, MDA and ROS levels, mPTP openness and the percentage of apoptotic cells	Doxorubicin-caused cardiotoxicity	Doxorubicin-treated cardiomyocytes
	Luteoloside [99]	Decreases levels of lactate dehydrogenase, ROS species, mPTP openness, caspase-3 activity, and apoptotic rate	I/R injury	H/R-induced I/R model in H9C2 cardiomyocytes
	Astragaloside IV [100]	Upregulates mitochondrial Bcl-2 expression, enhances antioxidant capacity, inhibits ROS, increases oxygen consumption, maintains *ΔΨ*m, and inhibits mPTP opening and apoptosis	I/R injury	H/R-treated H9C2 cells and anoxia/reoxygenation model in isolated rat heart
	Eriodictyol [101]	Suppresses the overload of intracellular Ca^2+^, prevents the overproduction of ROS, blocks mPTP opening, increases the *ΔΨ*m level, and decreases ATP depletion and upregulates Bcl-2 expression and downregulates Bax and caspase-3 expression	Myocardial infarction	H/R-induced I/R model in H9C2 cardiomyocytes
	Dihydromyricetin [102]	Increases ATP content, mitochondrial DNA content, and citrate synthase activity and decreases ROS level, mitochondrial MnSOD activity, and caspase-3 activity	I/R injury	I/R model in mice and H/R-treated cardiomyocytes from mice
	Vitexin [103]	Reduces ROS levels; improves mitochondrial activity, *ΔΨ*m, and ATP content; increases mfn2 expression, and reduces the recruitment of Drp1 in the mitochondria	I/R injury	I/R model in isolated rat heart and H/R-induced I/R model in H9C2
	Honokiol [104]	Inhibits ROS production and regulates *ΔΨ*m.	I/R injury	I/R model in C57BL/6 mice and H/R-treated cardiomyocytes from neonatal rats
	Apigenin [105]	Reduces the activity of lactate dehydrogenase and intracellular ROS, alleviates the loss of *ΔΨ*m, prevents mPTP opening, and decreases caspase-3 activity, cytochrome c release, and apoptosis	I/R injury	I/R model in isolated rat heart and ischemic/reperfusion medium-induced injury model in cardiomyocytes

**Table 8:** Similarities and differences between the mitochondrial mechanisms for natural products regulating different diseases.

Diseases	Major mechanisms	Natural products
Cancer	Energy metabolism obstruction	Rhein [39]
		Gracillin [44]
	Oxidative stress	*Bulbine frutescens* [37]
		Bullfrog oil [38]
		Orientin [40]
		Asparanin A [42]
		Gracillin [44]
	Apoptosis	*Bulbine frutescens* [37]
		Orientin [40]
		Licochalcone A [41]
		Asparanin A [42]
		Parameritannin A-2 [43]
		Cernumidine [45]
	Mitochondrial membrane potential imbalance	Bullfrog oil [38]
		Rhein [39]
		Asparanin A [42]
		Cernumidine [45]

Neurodegenerative diseases	Energy metabolism obstruction	*Solanum melongena* extract [46]
		*Ganoderma lucidum* [47]
		*α*-Arbutin [51]
		Auraptene [54]
	Oxidative stress	*Solanum melongena* extract [46]
		Linalool [48]
		*α*-Arbutin [51]
		Naringenin [52]
		Apigenin [53]
		Auraptene [54]
	Apoptosis	*Ganoderma lucidum* [47]
		Cinnamic acid derivatives [49]
		Proanthocyanidins [50]
		Naringenin [52]
		Apigenin [53]
		Naringenin [55]
		Ulmoside A [56]
	Mitochondrial membrane potential imbalance	*Ganoderma lucidum* [47]
		Linalool [48]
		*α*-Arbutin [51]
	Mitochondrial fusion, division, and autophagy	Celastrol [57]

Liver diseases	Energy metabolism obstruction	Rooibos tea [58]
		*Cimicifuga racemosa* extract [59]
		*Polygonatum kingianum* [61]
		Betaine [63]
		Nicotinamide riboside [64]
		Puerarin [65]
		*Punica granatum* L. [62]
		Silybin [67]
		Salvianolic acid B [68]
	Oxidative stress	*Cimicifuga racemosa* extract [59]
		Sipjeondaebo-tang [60]
		*Polygonatum kingianum* [61]
		Nicotinamide riboside [64]
		Diosgenin [66]
		Silybin [67]
		NecroX-7 [69]
	Apoptosis	*Cimicifuga racemosa* extract [59]
		*Polygonatum kingianum* [61]
		Betaine [63]
		Silybin [67]
		Salvianolic acid B [68]
	Mitochondrial membrane potential imbalance	*Cimicifuga racemosa* extract [59]
		Sipjeondaebo-tang [60]
		Diosgenin [66]
		Salvianolic acid B [68]
		NecroX-7 [69]
	Fatty acid oxidation	Silybin [67]
		NecroX-7 [69]

T2DM	Energy metabolism obstruction	Polysaccharides from *Portulaca oleracea* L. [70]
		Korean red ginseng [71]
		Berberine [72]
		Quercetin [73]
		Theaflavins [74]
		Puerarin [76]
	Mitochondrial membrane potential imbalance	Polysaccharides from *Portulaca oleracea* L. [70]
		Silibinin [75]
	Apoptosis	Silibinin [75]
	Mitochondrial fusion, division, and autophagy	Korean red ginseng [71]
		Quercetin [73]
		Theaflavins [74]
		Silibinin [75]
		Puerarin [76]

Diabetes complications	Energy metabolism obstruction	QiDiTangShen granules [77]
		Shengmai San [78]
		Water extracts of Liuwei Dihuang [79]
		Salidroside [82]
		Hydroxytyrosol [85]
	Oxidative stress	Water extracts of Liuwei Dihuang [79]
		Anthocyanins [80]
		Ginsenoside Rb1 [84]
		Hydroxytyrosol [85]
	Apoptosis	Anthocyanins [80]
		Orientin [81]
		Astragalus polysaccharides [83]
		Ginsenoside Rb1 [84]
	Mitochondrial membrane potential imbalance	Orientin [81]
		Water extracts of Liuwei Dihuang [79]
	Mitochondrial fusion, division, and autophagy	Orientin [81]
		QiDiTangShen granules [77]
		Salidroside [82]

Obesity	Energy metabolism obstruction	Melinjo (*Gnetum gnemon* L.) seed extract [88]
		*Cinnamomum cassia* Presl [89]
		Isorhamnetin [91]
		Zeaxanthin [92]
		Berberine [93]
		Purpurin [94]
		Epigallocatechin-3-gallate [95]
		Guarana (*Paullinia cupana* Kunth) [90]
	Mitochondrial membrane potential imbalance	Zeaxanthin [92]
		Berberine [93]
		Purpurin [94]
	Mitochondrial fusion, division, and autophagy	*Cinnamomum cassia* Presl [89]
		Isorhamnetin [91]
		Zeaxanthin [92]
		Epigallocatechin-3-gallate [95]
		Guarana (*Paullinia cupana* Kunth) [90]
	Fatty acid metabolism	Green tea [86]

Myocardial injury	Energy metabolism obstruction	Propolis [96]
		Luteoloside [99]
		Eriodictyol [101]
		Dihydromyricetin [102]
		Vitexin [103]
		Apigenin [105]
	Oxidative stress	Capsaicin [97]
		Quercetin [98]
		Luteoloside [99]
		Astragaloside IV [100]
		Eriodictyol [101]
		Dihydromyricetin [102]
		Vitexin [103]
		Honokiol [104]
		Apigenin [105]
	Apoptosis	Capsaicin [97]
		Quercetin [98]
		Luteoloside [99]
		Astragaloside IV [100]
		Eriodictyol [101]
		Dihydromyricetin [102]
		Apigenin [105]
	Mitochondrial membrane potential imbalance	Capsaicin [97]
		Quercetin [98]
		Luteoloside [99]
		Astragaloside IV [100]
		Eriodictyol [101]
		Vitexin [103]
		Honokiol [104]
		Apigenin [105]

## Data Availability

My article is a summary, so there is no data to provide.

## Abbreviations

AFLD: Alcoholic fatty liver disease
ASH: Alcoholic steatohepatitis
DM: Diabetes mellitus
H/R: Hypoxia/reoxygenation
I/R: Ischemia/reperfusion
IR: Insulin resistance
LPS: Lipopolysaccharides
*ΔΨ*m: Mitochondrial membrane potential
MPTP: 1-Methyl-4-phenyl-1,2,3,6-tetrahydropyridine
NAFLD: Nonalcoholic fatty liver disease
NASH: Nonalcoholic steatohepatitis
T1DM: Type 1 diabetes mellitus
T2DM: Type 2 diabetes mellitus.
